# Modified differential evolution algorithm with onlooker bee operator for mixed discrete-continuous optimization

**DOI:** 10.1186/s40064-016-3560-z

**Published:** 2016-11-03

**Authors:** Yongfei Miao, Qinghua Su, Zhongbo Hu, Xuewen Xia

**Affiliations:** 1 School of Computer Science and Technology, Wuhan University of Technology, Wuhan, 430070 People’s Republic of China; 2 School of Information and Mathematics, Yangtze University, Jingzhou, 434023 Hubei People’s Republic of China; 3 School of Software, East China Jiaotong University, Nanchang, 330013 People’s Republic of China

**Keywords:** Artificial bee colony algorithm, Design of coil spring problem, Differential evolution Algorithm

## Abstract

For solving non-linear programming problems containing discrete and continuous variables, this article suggests two modified algorithms based on differential evolution (DE). The two proposed algorithms incorporate a novel random search strategy into DE/best/1 and DE/cur-to-best/1 respectively. Inspired by the artificial bee colony algorithm, the random search strategy overcomes the searching unbalance of DE/best/1 and DE/cur-to-best/1 by enhancing the global exploration capability of promising individuals. Two numerical experiments are given to test the two modified algorithms. Experiment 1 is conducted on the benchmark function set of CEC2005 in order to verify the effectiveness of the improved strategy. Experiment 2 is designed to optimize two mixed discrete-continuous problems to illustrate the competitiveness and the practicality of the proposed algorithms. In particular, the modified DE/cur-to-best/1 finds the new optima of two engineering optimization problems.

## Background

Among the most commonly used stochastic algorithms, the differential evolution algorithm (DE) Storn and Price (1995) proposed by Storn and Price in 1995 has been identified as one of the most powerful optimizers. DE is the only such algorithm that has secured competitive ranking in all optimization competitions at IEEE International Conferences on Evolutionary Computation (CEC) (Das and Suganthan 2011; Elsayed et al. 2011; LaTorre et al. 2011) since 1996. The competitiveness of DE is also supported by many comparative studies (Civicioglu and Besdok 2013; Wang et al. 2011; Montes and MirandaVarela 2010; Vesterstrom and Thomsen 2004). However, there remains a shortfall in the search balance in the two mutation strategies, i.e., DE/best/1 and DE/cur-to-best/1, which are good at exploitation and poor at exploration. This often causes stagnation during the solution of complex problems.

Artificial bee colony (ABC), developed by Karaboga (2005), is a novel heuristic algorithm inspired by the foraging behavior of honey bee swarms. In ABC, a food source position represents a possible solution of the optimization problem and the amount of each food source represents its fitness. There is only one bee for each food source. The colony is classified into three groups depending on their duties: employed bees, onlooker bees and scouts. The number of employed and onlooker bees is equal to one half of the population size. Based on the information shared by employed bees, onlooker bees select different food sources at different probabilities and explore their neighborhood. Some numerical comparisons (Civicioglu and Besdok 2013; Karaboga and Basturk 2007, 2008, 2009) have demonstrated that the performance of the ABC algorithm is competitive with other population-based algorithms, and it has the advantage of employing fewer control parameters.

The evolutionary operators of the DE and ABC are similar and, in some ways, complementary. Some recent studies combining the two algorithms have been proposed to benefit from their advantages and overcome their drawbacks. Yang et al. (2013) proposed a hybrid ABC-DE algorithm, in which employed bees use the mutation and crossover strategies of DE to enforce their exploration ability, while onlooker bees keep their original updating strategy to retain the exploitation capability. Gao et al. (2012) proposed a modified ABC algorithm, which is based on the fact that each bee searches only around the best solution of the previous iteration. Gao (2013) gave an accelerated ABC algorithm based on DE for solving the Van der pol-Duffing oscillator problem. Álvaro et al. (2012) developed a multi-objective ABC/DE algorithm by combining the collective intelligence of the honey bee swarms with the properties of the DE algorithm. Many other successful combinations (Gao and Liu 2011; Li and Yin 2012; Li et al. 2013) of the two algorithms have also demonstrated complementarity of the operators in the searching ability.

In order to enhance the exploration ability of the DE/best/1 and DE/cur-to-best/1 mutation strategies, the present study proposes two modified DE algorithms with an onlooker bee operator, called mDEOB (i.e., mDEOB/best/1 and mDEOB/cur-to-best/1). Inspired by ABC, the two mDEOB algorithms run the classical mutation and crossover operators of DE followed by a random search guided by an onlooker bee operator. The random search enhances the ability to explore promising individuals. Two numerical experiments were conducted on the benchmark function set of CEC2005 and a class of engineering design problems. Statistical analyses and comparative analyses were performed on the results of the two experiments.

The rest of this paper is structured as follows: “Classical differential evolution” section briefly introduces the classical DE algorithm; “Modified differential evolution algorithm with onlooker bee operator” section presents and analyzes the proposed mDEOB algorithms; numerical experiments and analyses are then presented in “Numerical experiment” section, followed by conclusions in “Conclusion” section.

## Classical differential evolution

DE is often used for dealing with the continuous optimization problem. This paper supposes that the objective function to be minimized is $f({\vec {x}}),\ {\vec {x}}=(x_{1},\ldots ,x_{D})\in \mathfrak {R}^{D}$, and the feasible solution space is $\Psi =\prod _{j=1}^{j=D}[L_{j},U_{j}]$. The classical DE (Hu et al. 2008, 2013, 2014, 2016; Su and Hu 2013) works through a simple cycle of operators including mutation, crossover and selection operator after initialization. The classical DE procedures are described in detail as follows.

### Initialization

The first step of DE is the initialization of a population with *N* *D*-dimensional potential solutions (*individuals*) over the optimization search space. We shall symbolize each individual by ${\vec {x}}_{i}^{g}=(x_{i,1}^{g},x_{i,2}^{g},\ldots ,x_{i,D}^{g}),$ for $i=1,\ldots ,N,$ where $g=0,1,\ldots ,g_{max}$ is the current generation and $g_{max}$ is the maximum number of generations. For the first generation (*g* = 0), the population should be sufficiently scaled to cover the optimization search space as much as possible. Initialization is implemented by using a uniformly sampling to generate the potential individuals in the optimization search space. We can initialize the *j*th dimension of the *i*th individual according to

$$\begin{aligned} x_{i,j}^{0}=L_{j}+rand(0,1)\cdot (U_{j}-L_{j}) \end{aligned}$$

where *rand*(0, 1) is a uniformly distributed random number confined on the range [0,1].

### Mutation operator

After initialization, DE creates a *donor* vector ${\vec {v}}_{i}^{g}$ corresponding to each individual ${\vec {x}}_{i}^{g}$ in the *g*th generation through the mutation operator. This article is interested in the following two operators:

DE/best/1:

1 $$\begin{aligned} {\vec {v}}_{i}^{g}={\vec {x}}_{best}^{g}+F({\vec {x}}_{r_{1}}^{g}-{\vec {x}}_{r_{2}}^{g}); \end{aligned}$$

DE/current-to-best/1:

2 $$\begin{aligned} {\vec {v}}_{i}^{g}={\vec {x}}_{i}^{g}+F({\vec {x}}_{best}^{g}-{\vec {x}}_{i}^{g})+F({\vec {x}}_{r_{1}}^{g}-{\vec {x}}_{r_{2}}^{g}); \end{aligned}$$

where ${\vec {x}}_{best}^{g}$ denotes the best individual of the current generation, the indices $r_{1},r_{2}\in S_{r}=\{1,2,\ldots ,N\} \backslash$ *{i}* are uniformly random integers, mutually different and distinct from the sequential index *i*, and $F\in (0,1]$ is a real parameter, called *mutation* or *scaling factor*.

If the element values of the donor vector ${\vec {v}}_{i}$ exceed the pre-specified upper bound or lower bound, we can change the element values by the *periodic mode* rule as follow:

$$\begin{aligned} v_{i,j}=\left\{ \begin{array}{cc} U_j-(L_j-v_{i,j})\ \%\ |U_j-L_j| &\quad {\text {if}}\ v_{i,j}<L_j \\ L_j+(v_{i,j}-U_j)\ \%\ |U_j-L_j| &\quad {\text {if}}\ v_{i,j}>U_j \end{array}\right. \end{aligned}$$

### Crossover operator

Following mutation, the crossover operator is applied to further increasing the diversity of the population. In crossover, a trial vector, ${\vec {u}}_{i}^{g},$ is generated by the *binomial crossover*, which combines the elements of the target vectors, ${\vec {x}}_{i}^{g},$ and the donor vector, ${\vec {v}}_{i}^{g}.$

3 $$\begin{aligned} u_{i,j}^{g}=\left\{ \begin{array}{lll} v_{i,j}^{g} &\quad {\text {if}}\ rand(0,1)\le CR &\quad or\ j=j_{rand}\\ x_{i,j}^{g} &\quad {\text {otherwise}} \end{array}\right. \end{aligned}$$

where $CR\in (0,1)$ is the probability of crossover, $j_{rand}$ is a random integer on [1, *D*].

### Selection operator

Finally, the selection operator is employed to maintain the most promising trial individuals in the next generation. The classical DE adopts a simple selection scheme. It compares the objective value of the target ${\vec {x}}_{i}^{g}$ with that of the trial individual ${\vec {u}}_{i}^{g}$. If the trial individual reduces the value of the objective function, it is accepted for the next generation; otherwise the target individual is retained in the population. The selection operator is defined as

4 $$\begin{aligned} {\vec {x}}_{i}^{g+1}=\left\{ \begin{array}{ll} {\vec {u}}_{i}^{g}, &\quad {\text {if}}\ f({\vec {u}}_{i}^{g})<f({\vec {x}}_{i}^{g}) \\ {\vec {x}}_{i}^{g}, &\quad {\text {otherwise}}. \end{array}\right. \end{aligned}$$

## Modified differential evolution algorithm with onlooker bee operator

DE/best/1 and DE/cur-to-best/1 algorithms find the global optimum of simple optimization problems rapidly (e.g., low-dimensional convex optimization problems); however, both algorithms may easily become trapped in the local optima when solving complex multimodal problems, for the reason that exploration and exploitation capabilities are both necessary for a population-based optimizer. In fact, the exploration and exploitation requirements are mutually contradictory. In order to achieve a good performance, the two capabilities should be well balanced, but the solution search Eqs. (1) and (2), which are used to generate new candidate solutions, are based on the information of the current best solution. Thus the search ability of the algorithms is good at exploitation but poor at exploration.

**Fig. 1:**
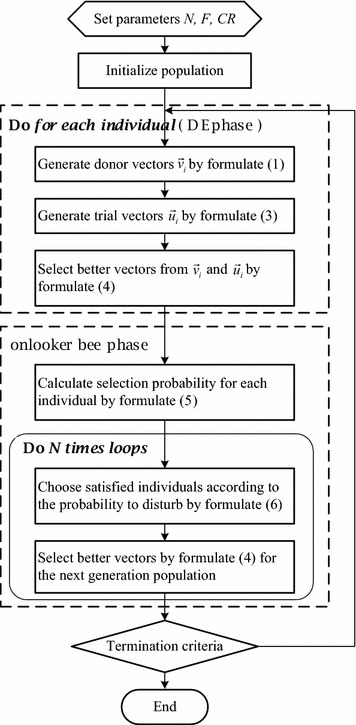
Flowchart of mDEOB/cur-to-best/1 algorithm. *Note* If replacing Eq. (2) with Eq. (1), the above flowchart represents the mDEOB/best/1 algorithm

In order to promote the balance of the exploitation and exploration capabilities, the present study adapts the ABC algorithm and proposes two modified differential evolution algorithms with an onlooker bee operator, called mDEOB (i.e., mDEOB/best/1 and mDEOB/cur-to-best/1). The mDEOB algorithms enhance DE’s exploration ability by adding some random searches around the promising individuals under the guidance of onlooker bees. The mDEOB algorithms work incorporate the two-stage cycle shown in Fig. 1, the first stage is the classical DE phase (including DE mutation, crossover and selection operators). The second stage is the onlooker bee phase inspired by the ABC algorithm. The implementation process of the onlooker bee phase is detailed below.

### Algorithmic flowchart

In the ABC algorithm, the artificial bee colony consists of three groups of bees: employed bees, onlookers and scout bees. Each employed bee exploits a food source, bring the information about the food source back to the hive and shares the information with onlooker bees waiting in the hive for this information. Each food source is a candidate solution of the problem. The amount of nectar in a food source represents the quality of the solution represented by the fitness value. An onlooker bee chooses a food source (candidate individual) depending on the probability value $P_i$ associated with the amount of nectar(fitness). The probability $P_i$ of the individual ${\vec {x}}_i$ is formulated as follows:

5 $$\begin{aligned} P_i\ =\ \frac{{\text {fitness}}({\vec {x}}_i)}{\sum ^N_{j=1}{\text {fitness}}({\vec {x}}_i)}. \end{aligned}$$

Note that the fitness should be changed appropriately when solving minimization problems. Here *N* denotes the number of food sources (population size).

Unlike the original ABC algorithm, the onlooker bees in mDEOB use the following Eq. (6) to generate new candidate solutions.

6 $$\begin{aligned} {\vec {y}}_{i}^{g}={\vec {x}}_{i}^{g}+F({\vec {x}}_{r_{1}}^{g}-{\vec {x}}_{r_{2}}^{g}). \end{aligned}$$

Obviously, Eq. (6) brings to the selected individual ${\vec {x}}_{i}^{g}$ a perturbation whose center is its own value and whose radius is the difference between two randomly selected individuals. The greedy selection operator [Eq. (4)] is used to decide whether ${\vec {x}}_{i}^{g}$ or ${\vec {y}}_{i}^{g}$ will survive to the population generated next. In each mDEOB cycle, *N* (population size) onlooker bees are sent to choose individuals in roulette wheel fashion: the greater the individual’s fitness, the greater its perturbation chance.

### Algorithmic analyses

**Fig. 2:**
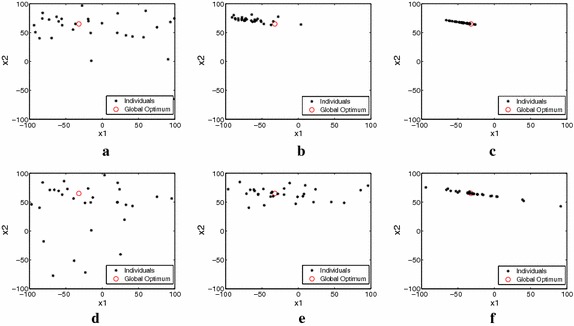
Population distribution observed at various stages of DE/best/1 and mDEOB/best/1. *Note* ‘FEs’ denotes the number of the function evaluations **a** DE: generation = 2, FEs = 60, **b** DE: generation = 4, FEs = 120, **c** DE: generation = 8, FEs = 240, **d** mDEOB: generation = 1, FEs = 60, **e** mDEOB: generation = 2, FEs = 120, **f** mDEOB: generation = 4, FEs = 240

Figure 2 illustrates how onlooker bees change the search process of the algorithms. Figure 2 shows the population distributions at various stages of DE/best/1 and mDEOB/best/1 when solving the third function of CEC2005.

In Fig. 2, it is readily seen that the population distributions have the following characteristics:

- For the same function evaluations (FEs), i.e., sub_figures (a) versus (d), (b) versus (e), (c) versus (f), the sub_figures (d), (e), (f) associated with mDEOB more diverse than those associated with DE.
- In the sub_figures (e) and (f), the candidate solutions are located on both sides of the global optimum, whereas they are located on one side in sub_figures (b) and (c).It is well known that the case where candidate solutions are distributed about the global optimum is more conducive to DE search. Thus, with the help of the onlooker bees, the mDEOB generates better more useful population distributions than DE alone.
- In sub_figure (e), many candidate solutions cover the global optimum and the population maintains better diversity; DE/best/1 (shown in sub_figure (c)) fails to do so. The figures illustrate the process by which this population diversity expedites more accurate solutions than is achievable using DE.

In summary, the numerical experiment results indicate that the modified strategy enhances the diversity of evolving populations, leading to improved of global searching in the solution space. This mitigates to some extent the disadvantages caused by the search imbalance in the DE/best/1 and DE/cur-to-best/1 algorithm.

Set parameters: population size $N = 30$, problem dimension $D = 2$, mutation factor $F = 0.5$, crossover probability $CR = 0.9$. The global optimum of the third function (2-dimension) of CEC2005 is ${\vec {x}} = (-32.2013,\ 64.9776)$ with the function value $f({\vec {x}}) = 0$.

## Numerical experiment

In order to test mDEOB/best/1 and mDEOB/cur-to-best/1 and show their performance, two numerical experiments are given in this section. One is conducted on the benchmark function set of CEC2005, the other is a group of application-oriented numerical examples related to two mixed discrete-continuous optimizations.

### Experiment 1: test on CEC2005

This subsection deals with the following tasks:

- Two comparative experiments on the benchmark function set of CEC2005 are conducted for mDEOB/best/1 versus DE/best/1 and mDEOB/cur-to-best/1 versus DE/cur-to-best/1.
- Sign Tests on the experimental results are used to demonstrate the advantage of mDEOB algorithms.
- Convergence figures on the first 14 benchmark functions, which include all functions except for 11 hybrid composition functions, are given to show the difference of convergence speed on the differential algorithms.

#### Designing experiments

The numerical experiments are conducted on 25 test instances proposed in the CEC2005 special session on real-parameter optimization Suganthan et al. (2005). The benchmark function set can be divided into four classes:

- 5 unimodal functions f1–f5;
- 7 basic multimodal functions f6–f12;
- 2 expanded multimodal functions f13–f14;
- 11 hybrid composition functions f15–f25.

The number of decision variables, *D*, is set to be 10 for all the 25 benchmark functions. The population size, *N*, is set to be 60 for all the algorithms. The mutation factor, *F*, is set to be 0.5 while the crossover probability, *CR*, is set to be 0.9. For each algorithm and each test function, 25 independent runs are conducted with 150000 function evaluations (FEs) as the termination criterion.

**Table 1:** Experimental results of mDEOB_best/1, DE/best/1 over 25 runs on 25 test functions with 150000 FEs

	DE	mDEOB	DE	mDEOB	DE	mDEOB	DE	mDEOB	DE	mDEOB
	f1		f2		f3		f4		f5	
1st	0.00E+00	0.00E+00	0.00E+00	0.00E+00	2.27E−13	0.00E+00	0.00E+00	0.00E+00	4.99E−07	0.00E+00
7th	0.00E+00	0.00E+00	0.00E+00	0.00E+00	2.22E−12	0.00E+00	0.00E+00	0.00E+00	8.51E−07	0.00E+00
13th	0.00E+00	0.00E+00	0.00E+00	0.00E+00	4.32E−12	0.00E+00	0.00E+00	0.00E+00	1.21E−06	0.00E+00
19th	0.00E+00	0.00E+00	0.00E+00	0.00E+00	8.92E−12	0.00E+00	0.00E+00	0.00E+00	2.00E−06	0.00E+00
25th	0.00E+00	0.00E+00	0.00E+00	0.00E+00	2.00E−11	0.00E+00	0.00E+00	0.00E+00	4.08E−06	5.46E−12
Mean	0.00E+00	0.00E+00	0.00E+00	0.00E+00	6.21E−12	0.00E+00	0.00E+00	0.00E+E+00	1.57E−06	3.64E−13
SD	0.00E+00	0.00E+00	0.00E+00	0.00E+00	5.44E−12	0.00E+00	0.00E+00	0.00E+00	9.81E−07	1.26E−12
Compare	$\approx$		$\approx$		+		$\approx$		+	
	f6		f7		f8		f9		f10	
1st	0.00E+00	0.00E+00	3.79E−01	7.40E−03	2.02E+01	2.01E+01	1.40E+01	3.98E+00	2.00E+01	1.99E+00
7th	0.00E+00	0.00E+00	4.60E−01	5.16E−02	2.05E+01	2.03E+01	1.74E+01	5.97E+00	2.80E+01	6.96E+00
13th	0.00E+00	0.00E+00	4.93E−01	6.64E−02	2.05E+01	2.04E+01	2.04E+01	7.96E+00	3.03E+01	1.09E+01
19th	0.00E+00	0.00E+00	5.38E−01	1.21E−01	2.05E+01	2.05E+01	2.57E+01	1.19E+01	3.12E+01	1.79E+01
25th	0.00E+00	3.99E+00	6.16E−01	2.58E−01	2.07E+01	2.06E+01	2.99E+01	2.09E+01	3.93E+01	2.39E+01
Mean	0.00E+00	6.38E−01	4.98E−01	8.71E−02	2.05E+01	2.04E+01	2.12E+01	8.99E+00	2.98E+01	1.23E+01
SD	0.00E+00	1.46E+00	6.02E−02	5.83E−02	9.78E−02	1.26E−01	4.52E+00	4.09E+00	4.11E+00	6.07E+00
Compare	−		+		+		+		+	
	f11		f12		f13		f14		f15	
1st	7.57E+00	8.17E−03	0.00E+00	0.00E+00	1.87E+00	3.37E−01	3.34E+00	1.67E+00	1.54E+02	8.99E+01
7th	8.79E+00	1.79E+00	0.00E+00	0.00E+00	2.08E+00	6.21E−01	3.57E+00	2.24E+00	2.09E+02	1.46E+02
13th	8.95E+00	2.75E+00	0.00E+00	0.00E+00	2.36E+00	8.33E−01	3.69E+00	2.50E+00	2.44E+02	1.97E+02
19th	9.26E+00	3.47E+00	2.27E−13	1.88E+01	2.56E+00	1.02E+00	3.79E+00	3.03E+00	2.64E+02	4.27E+02
25th	9.93E+00	5.38E+00	1.00E+01	1.69E+03	2.80E+00	1.91E+00	3.96E+00	3.92E+00	4.86E+02	6.03E+02
Mean	8.91E+00	2.63E+00	8.00E−01	1.54E+02	2.31E+00	8.72E−01	3.68E+00	2.58E+00	2.52E+02	2.71E+02
SD	5.54E−01	1.29E+00	2.71E+00	4.29E+02	2.84E−01	3.49E−01	1.56E−01	5.10E−01	7.29E+01	1.50E+02
Compare	+		−		+		+		+	
	f16		f17		f18		f19		f20	
1st	1.33E+02	9.81E+01	1.47E+02	9.49E+01	3.00E+02	6.00E+02	8.00E+02	7.90E+02	3.00E+02	7.90E+02
7th	1.54E+02	1.06E+02	1.67E+02	1.06E+02	8.00E+02	8.00E+02	8.00E+02	8.00E+02	8.00E+02	8.00E+02
13th	1.62E+02	1.20E+02	1.74E+02	1.14E+02	8.00E+02	9.30E+02	8.00E+02	9.17E+02	8.00E+02	9.17E+02
19th	1.67E+02	1.34E+02	1.80E+02	1.22E+02	8.00E+02	9.58E+02	8.00E+02	9.47E+02	8.00E+02	9.47E+02
25th	1.77E+02	1.65E+02	1.98E+02	1.42E+02	8.00E+02	9.99E+02	8.00E+02	1.00E+03	8.00E+02	1.01E+03
Mean	1.60E+02	1.22E+02	1.73E+02	1.16E+02	7.80E+02	8.93E+02	8.00E+02	8.93E+02	7.80E+02	8.97E+02
SD	1.05E+01	1.76E+01	1.12E+01	1.33E+01	9.80E+01	9.16E+01	0.00E+00	7.57E+01	9.80E+01	7.43E+01
Compare	+		+		−		+		−	
	f21		f22		f23		f24		f25	
1st	3.00E+02	3.00E+02	3.00E+02	7.25E+02	5.59E+02	5.59E+02	2.00E+02	2.00E+02	2.00E+02	2.00E+02
7th	5.00E+02	5.00E+02	7.69E+02	7.37E+02	5.59E+02	7.29E+02	2.00E+02	2.00E+02	2.00E+02	2.00E+02
13th	5.00E+02	9.94E+02	7.71E+02	7.45E+02	5.59E+02	9.71E+02	2.00E+02	2.00E+02	2.00E+02	2.00E+02
19th	5.00E+02	1.11E+03	7.73E+02	7.64E+02	5.59E+02	1.15E+03	2.00E+02	2.00E+02	2.00E+02	2.00E+02
25th	5.00E+02	1.18E+03	7.77E+02	8.70E+02	7.21E+02	1.22E+03	2.00E+02	1.25E+03	2.00E+02	1.25E+03
Mean	4.68E+02	8.27E+02	7.52E+02	7.62E+02	5.85E+02	9.59E+02	2.00E+02	3.14E+02	2.00E+02	3.14E+02
SD	7.33E+01	3.29E+02	9.23E+01	4.01E+01	5.93E+01	2.26E+02	0.00E+00	2.44E+02	0.00E+00	2.44E+02
Compare	−		−		−		−		−	

‘$+,\approx ,-$’ denote that the results of mDEOB/best/1 are ‘better’, ‘approximate’ and ‘worse’ than the corresponding DE, respectively

**Table 2:** Experimental results of mDEOB/cur-to-best/1, DE/cur-best/1 over 25 runs on 25 test functions with 150,000 FEs

	DE	mDEOB	DE	mDEOB	DE	mDEOB	DE	mDEOB	DE	mDEOB
	f1		f2		f3		f4		f5	
1st	0.00E+00	0.00E+00	5.50E−07	0.00E+00	4.89E+02	0.00E+00	2.27E−13	0.00E+00	1.28E+02	0.00E+00
7th	8.55E−05	0.00E+00	1.74E−02	0.00E+00	1.90E+03	0.00E+00	9.29E−08	0.00E+00	3.53E+02	0.00E+00
13th	2.28E−03	0.00E+00	6.37E−01	0.00E+00	1.15E+04	0.00E+00	4.12E−05	0.00E+00	9.36E+02	0.00E+00
19th	2.60E−02	0.00E+00	2.64E+01	0.00E+00	2.98E+04	0.00E+00	1.69E−03	0.00E+00	1.50E+03	0.00E+00
25th	1.14E+01	0.00E+00	6.33E+02	0.00E+00	1.71E+05	0.00E+00	4.06E−02	0.00E+00	3.65E+03	0.00E+00
Mean	7.90E−01	0.00E+00	6.52E+01	0.00E+00	2.53E+04	0.00E+00	3.34E−03	0.00E+00	1.14E+03	0.00E+00
SD	2.54E+00	0.00E+00	1.47E+02	0.00E+00	3.98E+04	0.00E+00	8.70E−03	0.00E+00	8.95E+02	0.00E+00
Compare	+		+		+		+		+	
	f6		f7		f8		f9		f10	
1st	3.99E+00	0.00E+00	1.72E−02	7.40E−03	2.01E+01	2.02E+01	0.00E+00	2.29E−02	3.98E+00	3.02E+00
7th	8.35E+00	0.00E+00	7.14E−02	2.06E−02	2.02E+01	2.02E+01	3.98E+00	1.03E+00	7.96E+00	7.69E+00
13th	1.98E+01	0.00E+00	3.13E−01	3.45E−02	2.02E+01	2.03E+01	4.97E+00	2.50E+00	1.19E+01	9.61E+00
19th	6.73E+01	0.00E+00	3.64E−01	5.66E−02	2.03E+01	2.03E+01	6.17E+00	4.32E+00	1.29E+01	1.28E+01
25th	5.70E+03	3.99E+00	2.32E+00	3.32E−01	2.04E+01	2.04E+01	1.29E+01	8.25E+00	1.99E+01	2.41E+01
Mean	2.77E+02	4.78E−01	4.98E−01	5.40E−02	2.02E+01	2.03E+01	5.30E+00	3.24E+00	1.13E+01	1.04E+01
SD	1.11E+03	1.30E+00	6.13E−01	6.40E−02	7.59E−02	7.74E−02	2.87E+00	2.16E+00	3.85E+00	4.42E+00
Compare	+		+		−		−		+	
	f11		f12		f13		f14		f15	
1st	3.52E−02	3.21E−05	0.00E+00	0.00E+00	2.80E−01	9.20E−01	1.10E+00	1.59E+00	0.00E+00	0.00E+00
7th	3.71E−01	8.40E−02	1.00E+01	0.00E+00	5.42E−01	1.29E+00	1.76E+00	2.36E+00	7.81E+01	5.21E+01
13th	1.34E+00	3.44E+00	1.52E+01	0.00E+00	7.12E−01	1.64E+00	2.30E+00	2.56E+00	1.36E+02	7.25E+01
19th	2.13E+00	6.68E+00	7.12E+02	1.00E+01	7.83E−01	1.81E+00	2.55E+00	2.65E+00	4.25E+02	1.30E+02
25th	3.11E+00	8.41E+00	2.13E+03	1.56E+03	1.25E+00	2.27E+00	3.08E+00	3.01E+00	6.21E+02	4.10E+02
Mean	1.31E+00	3.49E+00	4.31E+02	6.65E+01	7.23E−01	1.58E+00	2.14E+00	2.49E+00	2.60E+02	1.17E+02
SD	9.58E−01	2.88E+00	7.05E+02	3.04E+02	2.41E−01	3.35E−01	5.17E−01	2.90E−01	2.12E+02	1.13E+02
Compare	+		+		−		−		+	
	f16		f17		f18		f19		f20	
1st	9.27E+01	5.97E+01	9.37E+01	1.00E+02	8.00E+02	3.00E+02	8.00E+02	5.01E+02	8.00E+02	3.00E+02
7th	1.05E+02	9.62E+01	1.02E+02	1.08E+02	8.01E+02	8.00E+02	8.01E+02	8.00E+02	8.01E+02	8.00E+02
13th	1.14E+02	1.03E+02	1.14E+02	1.23E+02	9.21E+02	8.00E+02	9.21E+02	8.00E+02	9.21E+02	8.00E+02
19th	1.26E+02	1.14E+02	1.27E+02	1.35E+02	9.76E+02	8.00E+02	9.88E+02	9.06E+02	9.89E+02	8.82E+02
25th	1.48E+02	1.26E+02	1.64E+02	1.48E+02	1.07E+03	9.37E+02	1.03E+03	9.50E+02	1.03E+03	9.51E+02
Mean	1.16E+02	1.05E+02	1.15E+02	1.21E+02	9.08E+02	7.45E+02	9.09E+02	8.26E+02	9.05E+02	8.01E+02
SD	1.41E+01	1.41E+01	1.76E+01	1.45E+01	8.64E+01	1.76E+02	8.94E+01	8.69E+01	8.55E+01	1.28E+02
Compare	+		−		+		+		+	
	f21		f22		f23		f24		f25	
1st	3.00E+02	3.00E+02	3.01E+02	3.00E+02	5.59E+02	5.59E+02	2.00E+02	2.00E+02	2.00E+02	2.00E+02
7th	8.75E+02	3.00E+02	7.35E+02	7.56E+02	9.71E+02	7.21E+02	2.00E+02	2.00E+02	2.00E+02	2.00E+02
13th	9.71E+02	3.00E+02	7.55E+02	7.61E+02	1.08E+03	7.21E+02	2.01E+02	2.00E+02	2.01E+02	2.00E+02
19th	1.13E+03	5.00E+02	8.00E+02	7.64E+02	1.18E+03	9.71E+02	5.41E+02	2.00E+02	5.41E+02	2.00E+02
25th	1.20E+03	9.00E+02	9.63E+02	8.00E+02	1.26E+03	1.09E+03	1.23E+03	7.00E+02	1.23E+03	7.00E+02
Mean	9.04E+02	4.34E+02	7.57E+02	7.30E+02	1.03E+03	8.21E+02	4.65E+02	2.44E+02	4.65E+02	2.44E+02
SD	2.86E+02	1.96E+02	1.09E+02	1.28E+02	1.88E+02	1.75E+02	3.35E+02	1.24E+02	3.35E+02	1.24E+02
Compare	+		+		+		+		+	

**Fig. 3:**
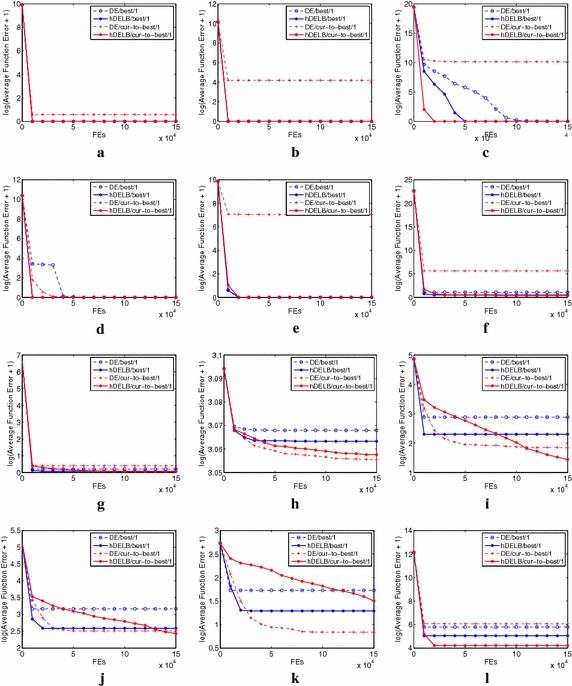
Evolution figures of the average error of the best function values on 25 running derived from mDEOB/best/1, mDEOB/cur_to_best/1, and two corresponding DE algorithms **a** f1, **b** f2, **c** f3, **d** f4, **e** f5, **f** f6, **g** f7, **h** f8, **i** f9, **j** f10, **k** f11, **l** f12

**Table 3:** Sign test of experimental results in Tables 1 and 2

	In Table 1					In Table 2				
	Neg. Dif.	Pos. Dif.	Tie	Total	*P* value	Neg. Dif.	Pos. Dif.	Tie	Total	*P* value
On best value	3	13	9	25	0.021	6	13	6	25	0.167
On mean value	11	11	3	25	1.000	5	20	0	25	0.004

‘Neg. Dif.’ and ‘Pos. Dif.’ denote the number of the negative and positive differences, respectively ‘*P* value’ denotes the probability value supporting the null hypothesis

#### Statistical analysis of experimental results

According to Suganthan et al. (2005), Table 1 reports seven results of 25 independent runs on each function by DE/best/1 and mDEOB/best/1: the minimal error of 25 runs, the 7th error, the 13th error, the 19th error, the maximal error, the average error (mean) and the standard deviation (std.) of 25 runs, in turn. In the “compare” row, simple comparison analyses are given. The priority of the comparison analyses is the best solution, the mean and the standard deviation in turn. Table 2 reports the similar results for DE/cur-to-best/1 and mDEOB/cur-to-best/1. From Table 1, we can see that mDEOB/best/1 outperforms the DE/best/1 on the 5 unimodal functions and 9 multimodal functions. Especially, as shown in Table 2, mDEOB/cur-to-best/1 outperforms the DE/cur-to-best/1 on all unimodal functions and all hybrid composition functions except for f17. mDEOB/cur-to-best/1 is superior to DE/cur-to-best/1 on the 5 functions (i.e., f6, f7, f10, f11 and f12) among the other 9 multimodal functions.

Sign Test Derrac et al. (2011) is a popular statistical method to compare the performances of algorithms. As we all known, the average error (mean) and the best value are two most important factors for the performances of algorithms. So this paper uses Sign Test method to analyze the average errors and the best values, which are shown in Tables 1 and 2. Here the null hypothesis is that the performances of the two algorithms are not significantly differential, while the alternative hypothesis is that the performances are clearly differential. As shown in Table 3, for the results in Table 1, the probability value of supporting the null hypothesis of Sign Test on the average errors equals 1.000, but the probability on the best values is 0.021, which is less than the significance level 0.05. That is to say, we cannot reject the null hypothesis according to the average errors, but we can reject the null hypothesis according to the best values. This indicates that (1) judging by the average errors, the performances of the two algorithms are not significantly differential, but (2) judging by the best values, the performances of the two algorithms are obviously differential. So the overall performance of mDEOB/best/1 algorithm is obviously differential with the other. Combining with the front “compare” rows in Table  1, we can then draw a conclusion that the overall performance of mDEOB/best/1 is better than DE/best/1. For the results in Table 2, the probability values of supporting the null hypothesis of Sign Test on the average errors and on the best values are equal to 0.004 and 0.167 respectively. In the similar way, we may draw a conclusion that mDEOB/cur-to-best/1 outperforms DE/cur-to-best/1.

Figure 3 shows the evolution landscapes of the average error of the best function values on 25 running derived from all the four algorithms on all unimodal functions and all basic multimodal functions (i.e., f1–f12). It is not difficult to find the overall superiority of mDEOB algorithms at the convergence speed.

In summary: we can get that onlooker bees strategy has positive effect on the performance of DE/best/1 and DE/cur-to-best/1.

### Experiment 2: mixed discrete-continuous

In order to further illustrate the capabilities of the proposed algorithms, two mixed discrete-continuous optimization problems (i.e., Design of a coil spring and a speed reducer) are optimized here.

#### Design of a coil spring

**Fig. 4:**
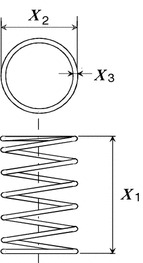
Spring design

The design of a coil spring Sandgren (1990), Shen et al. (2009) is a nonlinear engineering design optimization problem, which is designed to minimize the the volume of spring steel wire used to manufacture the spring (minimum weight). As shown in Fig. 4, the spring is to be a helical compression spring. The designing parameters are the number of spring coils, $x_1$, the outside diameter of the spring, $x_2$, and the spring wire diameter, $x_3$. Let $f(x_1,x_2,x_3)$ denote the objective function. The problem is formulated as follows:

$$\begin{aligned}&{\text {min.}}\ f(x_1,x_2,x_3) = \frac{\pi ^2 (x_1+2) x_2 x_3^2}{4} \\&{\text {s.t.}}\ g_1 (x_1,x_2,x_3) = \frac{8C_{\text {f}} F_{\text {max}} x_2}{\pi x_3^3} - S \le 0 \\&\quad \ g_2 (x_1,x_2,x_3) = l_{\text {f}} -l_{\text {max}} \le 0 \\&\quad \ g_3 (x_1,x_2,x_3) = d_{\text {min}} - x_3 \le 0 \\&\quad \ g_4 (x_1,x_2,x_3) = x_2 - D_{\text {max}} \le 0 \\&\quad \ g_5 (x_1,x_2,x_3) = 3.0 - \frac{x_2}{x_3}\le 0 \\&\quad \ g_6 (x_1,x_2,x_3) = \sigma _{\text {p}} - \sigma _{\text {pm}} \le 0 \\&\quad \ g_7 (x_1,x_2,x_3) = \sigma _{\text {p}} + \frac{F_{\text {max}} - F_{\text {p}}}{K} \\&\quad \qquad + 1.05(x_1 +2)x_3 - l_{\text {f}} \le 0 \\&\quad \ g_8 (x_1,x_2,x_3) = \sigma _{\text {w}}-\frac{F_{\text {max}} - F_{\text {p}}}{K}\le 0 \\&{\text {and}}\ 1 \le x_1 \le \frac{l_{\text {max}}}{d_{\text {min}}} \\&\quad \ 3d_{\text {min}} \le x_2 \le D_{\text {max}} \\&\quad \ d_{\text {min}} \le x_3 \le \frac{D_{\text {max}}}{3} \\&{\text {where}}\ C_{\text {f}} = \frac{4(x_2/x_3)-1}{4(x_2/x_3)-4} + \frac{0.615x_3}{x_2} \\&\quad \ K = \frac{G x_3^4}{8x_1 x_2^3} \\&\quad \sigma _{\text {p}} = \frac{F_{\text {p}}}{K} \\&\quad \ l_{\text {f}} = \frac{F_{\text {max}} }{K} + 1.05(x_1 +2)x_3 \end{aligned}$$

Here the above formula includes nine constants: $F_{\text {max}}=1000.0$, $S=189{,}000.0$, $l_{\text {max}}=14.0$, $d_{\text {min}}=0.2$, $D_{\text {max}}=3.0$, $F_{\text {p}}=300.0$, $\sigma _{\text {pm}}=6.0$, $\sigma _{\text {w}}=1.25$, $G=11.5\times 10^6$. $x_1$ is an integer variable, $x_2$ is a continuous variable and $x_3$ may take on only discrete variables according to the available standard of the spring steel wire diameters. The detailed explanation about the coil spring design can be found in reference Sandgren (1990), Lampinen and Zelinka (1999).

As reference Lampinen and Zelinka (1999), the two proposed mDEOB algorithms employ the soft-constraint (penalty) approach to handle the constraint functions, and employ the *INT()* function to handle the integer variable. The algorithmic parameters are set as follows, $D=3$, $N=40$, $F=0.9$ and $CR=0.8$. 100 independent runs are conducted with 2650 times iterations as the termination criterion.

**Table 4:** Optimal solution for coil spring problem

	Branch-Bound	GA	Meta-GA	DE/rand	This article: $\cdot$ / $\cdot$ / 1			
	Sandgren (1990)	Chen and Tsao (1993)	Wu and Chow (1995)	Lampinen and Zelinka (1999)	DE/best	mDEOB/best	DE/cur_to_best	mDEOB/cur_to_best
$x_1$	10	9	9	9	9	9	9	10
$x_2$	1.180701	1.2287	1.227411	1.223041	1.22304	1.22304	1.22304	1.18104
$x_3$	0.283	0.283	0.283	0.283	0.283	0.283	0.283	0.283
$g_1$	54309	415.969	550.993	1008.8114	1006.92	1006.93	1006.93	5389.66
$g_2$	8.8187	8.9207	8.9624	8.94564	8.94562	8.94562	8.94562	8.64751
$g_3$	0.08298	0.08300	0.08300	0.083000	0.08300	0.08300	0.08300	0.08300
$g_4$	1.8193	1.7713	1.7726	1.77696	1.77696	1.77696	1.77696	1.81896
$g_5$	1.1723	1.3417	1.3371	1.32170	1.32170	1.32170	1.32170	1.17330
$g_6$	5.4643	5.4568	5.4585	5.46429	5.46427	5.46427	5.46427	5.46398
$g_7$	0.0	0.0	0.0	2.68 × 10^−16^	0.0	0.0	0.0	0.0
$g_8$	0.0	00174	0.0134	5.08 × 10^−16^	2.34 × 10^−7^	2.76 × 10^−9^	9.61 × 10^−8^	6.71 × 10^−4^
*f*(*x*)	2.7995	2.6709	2.6681	2.65856	2.65856	2.65856	2.65856	2.65856
S.A.	–	–	–	95.0%	69.0%	88.0%	90.0%	95.0%

‘S.A.’ is the percentage in multiple runs of successfully achieving the optimal value

 As shown in Table 4, the solutions of the coil spring problem are reported and compared with the results obtained by other researchers. From the table, we can draw conclusions as follows:

- Firstly, the proposed algorithms, mDEOB/best/1 and mDEOB/cur_to_best/1, can find the minimal objective value obtained in literatures. The best results obtained by other researchers in Table 4 is 2.65856. mDEOB/best/1 and mDEOB/cur_to_best/1 can also find the optimal solution with the average CPU times of 0.02578s and 0.02322s in 100 independent runs.
- Secondly, mDEOB/cur_to_best/1 finds another optimal solution. The find provides another designing strategy of the coil spring. From Table 4, the optimal solution reported by Lampinen et.al. is (9, 1.223041, 0.238) of $(x_1, x_2, x_3)$. DE/best, mDEOB/best/1 and DE/cur_to_best/1 algorithms find the same optimal solution, while mDEOB/cur_to_best/1 finds another optimal solution, (10, 1.18104, 0.283).
- Thirdly, the percentage of successfully achieving the optimal value in multiple runs demonstrates that the modified strategy of mDEOB is effective. The last row of Table  4 reports the percentage in 100 runs of successfully achieving the optimal value. The percentages of DE/best and DE/cur_to_best/1 are 69.0, 90.0% respectively, while those of the proposed mDEOB/best/1 and mDEOB/cur_to_best/1 are 88.0, 95.0%. This indicates that the robustness of mDEOB/best/1 and mDEOB/cur_to_best/1 algorithms are better than the corresponding DE algorithms. This improvement of algorithmic robustness could be only due to employing the onlooker bee operator.

#### Design of a speed reducer

Speed reducer design problem is a mixed programming problem containing one integer variable (i.e. the third variable $x_3$) and six continuous variables $x_i, i=1,2,\ldots ,7$, $(i\ne 3)$. The physical meaning of these variables can be seen in the reference Sadollah et al. (2013). There are eleven constraints resulting in the high complexity of the problem. Let ${\mathbf {x}}$ denote a vector $(x_1,x_2,x_3,x_4,x_5,x_6,x_7)$. The problem is formulated as follows:

$$\begin{aligned} {\text {min}}.\, {\text {f}}({\mathbf {x}} )&= 0.7854x_1x^2_2(3.3333x^2_3 + 14.933x_3 -43.0934) \\&\ \ \ -1.508x_1(x^2_6 + x^2_7) + 7.4777(x^3_6 + x^3_7) \\&\ \ \ + 0.7854(x_4x^2_6 + x_5x^2_7) \\ {\text {s.t.}}\ g_1 ({\mathbf {x}} )&= \frac{27}{x_1x^2_2x_3} - 1 \le 0 \\ g_2({\mathbf {x}} )&= \frac{397.5}{x_1x^2_2x^2_3} - 1 \le 0 \\ g_3({\mathbf {x}} )&= \frac{1.93x^3_4}{x_2x^4_6x_3} - 1 \le 0 \\ g_4({\mathbf {x}} )&= \frac{1.93x^3_5}{x_2x^4_7x_3} - 1 \le 0 \\ g_5({\mathbf {x}} )&= \frac{[(745(x_4/x_2x_3))^2 + 16.9 \times 10^6]^{1/2}}{110x^3_6} - 1 \le 0 \\ g_6({\mathbf {x}} )&= \frac{[(745(x_5/x_2x_3))^2 + 157.5 \times 10^6]^{1/2}}{85x^3_7} - 1 \le 0 \\ g_7({\mathbf {x}} )&= \frac{x_2x_3 }{40} - 1 \le 0 \\ g_8({\mathbf {x}} )&= \frac{5x_2 }{x_1} - 1\le 0 \\ g_9({\mathbf {x}} )&= \frac{x_1 }{12x_2} - 1\le 0 \\ g_{10}({\mathbf {x}} )&= \frac{1.5x_6 + 1.9}{x_4} - 1\le 0\\ g_{11}({\mathbf {x}} )&= \frac{1.1x_7 + 1.9}{x_5} - 1\le 0 \\ {\text {where}}&\ \ 2.6 \le x_1 \le 3.6,\ \ 0.7 \le x_2 \le 0.8, \\&\ \ 17 \le x_3 \le 28,\ \ 7.3 \le x_4, x_5 \le 8.3, \\&\ \ 2.9 \le x_6 \le 3.9,\ \ 5.0 \le x_7 \le 5.5 \end{aligned}$$

The reference Sadollah et al. (2013) reported the best results in current literatures, which are the results of six optimization methods including DELC, DEDS, PSO-DE, MDE, HEAA and MBA. We compare the results of the proposed mDEOB/cur_to_best algorithm with these best results. The comparative results are reported in Table  5. From the table, we can see that the proposed mDEOB/cur_to_best algorithm found a solution 2994.468551, which is better than others. This means that the solution of the proposed mDEOB/cur_to_best algorithm is the optimum in current literatures.

**Table 5:** Optimal solution for speed reducer design

	DELC	DEDS	HEAA	MDE	PSO-DE	MBA	mDEOB/cur_to_best
$x_1$	3.500000	3.500000	3.500022	3.500010	3.500000	3.500000	3.499998
$x_2$	0.700000	0.700000	0.70000039	0.70000	0.700000	0.700000	0.700000
$x_3$	17	17	17.000012	17	17.00000	17.00000	17.00000
$x_4$	7.300000	7.300000	7.300427	7.300156	7.300000	7.300033	7.300003
$x_5$	7.715319	7.715319	7.715377	7.80027	7.800000	7.715772	7.715313
$x_6$	3.35024	3.35024	3.350230	3.350221	3.350214	3.350218	3.350214
$x_7$	5.286654	5.286654	5.286663	5.286685	5.2866832	5.286654	5.286654
*f*(*x*)	2994.471066	2994.471066	2994.499107	2996.356689	2996.348167	2994.482453	2994.468551

In the constraint handling strategy and the handling method of integer variable $x_3$, the proposed mDEOB/cur_to_best algorithm respectively employs the soft-constraint (penalty) approach and the *INT()* function in reference Lampinen and Zelinka (1999) . The algorithmic parameters are set as follows, $D=7$, $N=50$, $F=0.9$ and $CR=0.8$. 100 independent runs are conducted with 2500 times iterations as the termination criterion.

In summary: The improved algorithms, especially mDEOB/cur_to_best algorithm, have strong competitiveness on this kind of complex constrained optimization problems.

All the above algorithms were implemented in Visual C++ and the experiments were conducted on a computer with a Intel(R) Xeon(R) CPU E3-1230 v3 @ 3.30GHz and 8GB RAM.

## Conclusion

Two new algorithms, mDEOB/best/1 and mDEOB/cur-to-best/1, are proposed to deal with the imbalance between exploration and exploitation capabilities of the DE/best/1 and DE/cur-to-best/1 algorithms. Inspired by the ABC algorithm, these offer improved exploration abilities by employing a random search guided by onlooker bees. Numerical experiments were conducted to test the two proposed algorithm on CEC2005 benchmark functions and two engineering optimization problems. The results on the CEC2005 benchmark functions indicated the effectiveness of the improved strategy. Comparison with other algorithms for the engineering optimization problems showed the competitiveness of the proposed algorithms. In particular, the mDEDE/cur-to-best/1 algorithm found the new optima in both problems.
